# Single-shot condensation of exciton polaritons and the hole burning effect

**DOI:** 10.1038/s41467-018-05349-4

**Published:** 2018-08-09

**Authors:** E. Estrecho, T. Gao, N. Bobrovska, M. D. Fraser, M. Steger, L. Pfeiffer, K. West, T. C. H. Liew, M. Matuszewski, D. W. Snoke, A. G. Truscott, E. A. Ostrovskaya

**Affiliations:** 1 ARC Centre of Excellence in Future Low-Energy Electronics Technologies and Nonlinear Physics Centre, Research School of Physics and Engineering, The Australian National University, Canberra, ACT 2601 Australia; 2 Institute of Molecular plus, Tianjin University, 300072 Tianjin, China; 3 Institute of Physics, Polish Academy of Sciences, A. Lotiników 32/46, 02-668 Warsaw, Poland; 4 JST, PRESTO, 4-1-8 Honcho, Kawaguchi, Saitama 332-0012 Japan; 5 Quantum Functional System Research Group, RIKEN Center for Emergent Matter Science, 2-1 Hirosawa, Wako-shi, Saitama 351-0198 Japan; 6 Department of Physics and Astronomy, University of Pittsburgh, Pittsburgh, PA 15260 USA; 7 Department of Electrical Engineering, Princeton University, Princeton, NJ 08544 USA; 8 Princeton Institute for the Science and Technology of Materials, Princeton University, Princeton, NJ 08544 USA; 9 Division of Physics and Applied Physics, School of Physical and Mathematical Sciences, College of Science, Nanyang Technological University, Singapore, 637371 Singapore; 10 Laser Physics Centre, Research School of Physics and Engineering, The Australian National University, Canberra, ACT 2601 Australia

## Abstract

A bosonic condensate of exciton polaritons in a semiconductor microcavity is a macroscopic quantum state subject to pumping and decay. The fundamental nature of this driven-dissipative condensate is still under debate. Here, we gain an insight into spontaneous condensation by imaging long-lifetime exciton polaritons in a high-quality inorganic microcavity in a single-shot optical excitation regime, without averaging over multiple condensate realisations. We demonstrate that condensation is strongly influenced by an incoherent reservoir and that the reservoir depletion, the so-called spatial hole burning, is critical for the transition to the ground state. Condensates of photon-like polaritons exhibit strong shot-to-shot fluctuations and density filamentation due to the effective self-focusing associated with the reservoir depletion. In contrast, condensates of exciton-like polaritons display smoother spatial density distributions and are second-order coherent. Our observations show that the single-shot measurements offer a unique opportunity to study fundamental properties of non-equilibrium condensation in the presence of a reservoir.

## Abstract (web summary)

The mechanism for exciton-polariton condensation in the presence of an incoherent reservoir has been long debated. Here the authors demonstrate the role of the spatial hole burning in condensation of long‐lived exciton polaritons by imaging the condensates in a single-shot excitation regime.

## Introduction

Exciton polaritons (polaritons herein) in semiconductor microcavities with embedded quantum wells (QWs)^1^ are composite bosonic quasiparticles that result from strong coupling between cavity photons and QW excitons, and exhibit a transition to quantum degeneracy akin to Bose–Einstein condensation (BEC)^2–7^. All signatures of BEC, such as macroscopic occupation of a ground state, long-range coherence, quantised circulation^8,9^, and superfluidity^10^, have been observed in this system in the past decade. However, due to the inherent driven-dissipative nature of the system stemming from the short lifetime of polaritons (from ~10^1^ to ~10^2^ ps) and the need to replenish them via optical or electrical pumping, the nature of the transition to the macroscopically occupied quantum state in polariton systems remains the subject of continuing debate. In particular, it has been conjectured that exciton polaritons exhibit a highly non-equilibrium Berezinskii–Kosterlitz–Thouless (BKT) rather than BEC phase^11–13^, while other studies support the assertion of exciton polariton condensation at thermodynamic equilibrium^14,15^. The difficulty in resolving the nature of condensation lies in the short time scales of the polariton dynamics. Continuous-wave (CW) regime experiments deal with a steady state reached by this driven-dissipative system, while pulsed experiments on polaritons are usually done by averaging over millions of realisations of the experiment. In both regimes, the time-integrated and ensemble-averaging imaging of the condensate by means of cavity photoluminescence spectroscopy washes out any dynamical and stochastic processes. For example, proliferation of spontaneously created dynamic phase defects (vortices) in the BKT phase transition^13^ cannot be unambiguously confirmed since only stationary vortices pinned by impurities or the lattice disorder potential survive the averaging process. To understand the process of polariton condensation and the evolution thereafter, one requires single-shot imaging of condensation dynamics.

Another difficulty in interpreting the experimental results lies in the strong influence of a reservoir of incoherent, high-energy excitonic quasiparticles on the condensation dynamics due to the spatial overlap between the reservoir and condensing polaritons. This overlap is particularly significant when the condensate is created by a Gaussian-shaped optical pump spot, which can localise both the long-lived excitonic reservoir and the short-lived polaritons in the gain region^16–19^. The interaction between the condensate and the reservoir particles is strong and repulsive, which enables creation of effective potentials by exploiting a local reservoir-induced energy barrier (blue shift)^20^ via a spatially structured optical pump. This technique has enabled observation of polariton condensates in a variety of optically induced trapping geometries^20–28^, as well as creation of condensates spatially separated from the pump (reservoir) region^20,29–32^. Despite the significant advances in creating and manipulating polariton condensates with the help of a steady-state reservoir induced by a CW pump, the influence of the reservoir on the condensate formation process is poorly understood. Recently, single-shot imaging performed on organic microcavities^33^ provided evidence in support of earlier theoretical suggestions that the reservoir is responsible for dynamical instability and subsequent spatial fragmentation of the polariton condensate in a wide range of excitation regimes^34–37^. Whether or not this behaviour is unique to organic materials, which are strongly influenced by material disorder, can only be determined by single-shot imaging in inorganic microcavities. Although single-shot experiments were previously performed in GaN and CdTe heterostructures^38,39^, single-shot real-space imaging of the condensate was thought impossible in inorganic microcavities due to insufficient brightness of the cavity photoluminescence.

In this work, we perform single-shot real-space imaging of exciton polaritons created by a short laser pulse in a high-quality inorganic microcavity supporting long-lifetime polaritons^15, 30,40^. By utilising the highly non-stationary single-shot regime, we show that the transition to ground-state condensation is driven primarily by reservoir depletion. This is in contrast to the quasi-stationary CW where this transition is driven by non-radiative (e.g. phonon-assisted^41^) energy relaxation processes that are more efficient for more excitonic polaritons^42^. Furthermore, we confirm that spatial fragmentation (filamentation) of the condensate density is an inherent property of a non-equilibrium, spontaneous bosonic condensation resulting from initial random population of high-energy and momenta states, and will persist even after relaxation to the lowest energy and momentum occurs. We unambiguously link this behaviour to the highly non-stationary nature of the condensate produced in a single-shot experiment, as well as to trapping of condensing polaritons in an effective random potential induced by spatially inhomogeneous depletion of the reservoir, i.e. the hole burning effect^34^. We argue that the reservoir depletion and the resulting filamentation is the feature of the condensate growth rather than an indication of its dynamical instability. Finally, we use a wide range of detuning between the cavity photon and QW excitons^40^ available in our experiments to vary the fraction of photon and exciton in a polariton quasiparticle^1,5^, and demonstrate transition from a condensate of light, photonic polaritons with strong filamentation and large shot-to-shot density fluctuations to a more homogeneous state of heavy, excitonic polaritons with reduced density fluctuations, which is only weakly affected by the incoherent reservoir.

## Results

### Transition to condensation

Spontaneous BEC of exciton polaritons is typically achieved with an optical pump which is tuned far above the exciton resonance in the microcavity^3^. The phonon-assisted and exciton-mediated relaxation of the injected free carriers^43^ then efficiently populates the available energy states of the lower polariton (LP) dispersion branch *E*(**k**), where **k** is the momentum in the plane of the QW. The reduced efficiency of the relaxation processes leads to accumulation of the polaritons in the bottleneck region at a high energy close to that of the exciton^6,44^. When stimulated scattering from this incoherent, high-energy excitonic reservoir into the **k** = 0 takes place, transition to condensation in the ground state of the LP dispersion *E*_min_(**k** = 0) is achieved^3,45^.

The transition to condensation in our experiment is driven by the far-off-resonant Gaussian pump with the spatial FWHM of ~25 μm. The pumping is performed by a sequence of short (~140 fs) laser pulses with 12.5 ns time interval between the pulses, which greatly exceeds both the condensate (~200 ps) and reservoir (~1 ns) lifetimes. A pulse picker (see Methods) picks out a single pulse in the sequence, effectively switching the pump off for 10 ms after the pulse. This ensures that a single realisation of a condensate is created and fully decayed, while the reservoir is not replenished. The cavity photoluminescence collected by the camera in the single-shot regime is therefore integrated over the entire lifetime (~200 ps) of the condensate (see Methods). When the experiment is performed without a pulse picker, each measurement is additionally ensemble-averaged over many realisations of the condensate.

**Fig. 1:**
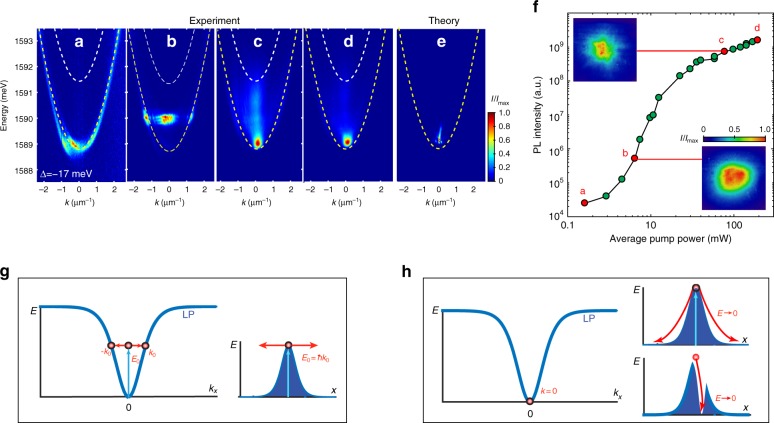
Signatures of transition to condensation. **a**–**d** Dispersion of lower polaritons *E*(*k* ≡ *k*_*x*_) ensemble-averaged over 10^6^ realisations of the pulsed experiment (**a**) below, **b** near and **c**, **d** above condensation threshold for the large negative exciton–photon detuning, Δ = −17 meV. Panel (**e**) shows the result of numerical modelling corresponding to the conditions in **d**. The white (yellow) dashed curves in **a**–**d** at higher (lower) energy correspond to the cavity photon (lower polariton), respectively. **f** Measurements of the ensemble-averaged emission intensity below and above condensation threshold. The emission is collected and integrated over a 70 × 70 μm detection window. Marked points correspond to the dispersion shown in **a**–**d**. Insets show ensemble-averaged real-space images of the polariton density below and above threshold in the detection window. **g** Schematics of the lower polariton dispersion curve, *E*(*k*_*x*_), and energy vs. position, *E*(*x*), for low excitation powers. The *E*(*x*) schematics illustrates ballistic expansion and condensation at *k*_*x*_ ≠ 0 corresponding to the dispersion shown in **b**. **h** Schematics of the lower polariton dispersion curve, *E*(*k*_*x*_), and energy vs. position, *E*(*x*), for high excitation powers. The *E*(*x*) schematics illustrate two possible routes to condensation at *k*_*x*_ = 0: energy relaxation with a non-depleted reservoir and reservoir depletion accompanied by energy relaxation

The polariton dispersion characterising the transition is shown in Fig. 1(a–d), where panels b–d demonstrate transition to condensation at *E*_min_(**k** = 0) with increasing rate of injection of the free carriers (optical pump power). Figure 1 is representative of the condensation process when the detuning between the cavity photon energy and the exciton resonance, Δ = *E*_c_ − *E*_ex_, is large and negative, i.e. for the polaritons that have a larger photonic fraction. Results for other values of detuning are presented in Supplementary Fig. 1 and Supplementary Fig. 2 of Supplementary Note 1. It should be noted that the images in Fig. 1(a–d) are ensemble-averaged over 10^6^ realisations of the polariton condensation experiment. In addition, time integration over the duration of the single-shot experiment should be taken into account when interpreting the *E*(*k*) in Fig. 1(a–d). Maxima of the photoluminescence intensity in these images correspond to the maxima of the polariton density, and the photoluminescence collected during the process of energy relaxation and decay of the condensate leads to smearing out of the image along the *E*-axis.

Near the condensation threshold, we observe the formation of a high-energy state shown in Fig. 1(b) characterised by a low density (as confirmed from low levels of photoluminescence intensity in Fig. 1(f)) and a flat dispersion. At the first glance, the transition from this highly non-equilibrium state at high energies to a **k** = 0 condensate at the bottom of the LP dispersion in Fig. 1(c, d) can be attributed entirely to energy relaxation processes. As has been previously demonstrated^42^ for negative detuning between cavity photon and exciton resonances, i.e. for the polaritons with a high photonic fraction, energy relaxation of polaritons down the LP branch is inefficient due to reduced scattering with phonons. Under CW excitation conditions, this leads to accumulation of polariton density in non-equilibrium metastable high-*k* states leading to stimulated bosonic scattering into these modes. In this ‘kinetic condensation’ regime, condensation into high-energy, high in-plane momenta states (**k** ≠ 0) is typically observed^46,47^. In contrast, in the regime of near-zero and positive detuning, Δ > 0, highly efficient phonon-assisted relaxation leads to efficient thermalisation and high mode occupations near the minimum of the LP branch *E*(**k**). Subsequently, condensation occurs into the ground state **k** = 0 assisted by stimulated bosonic scattering due to strong interactions of highly excitonic reservoir polaritons^42,47^.

**Fig. 2:**
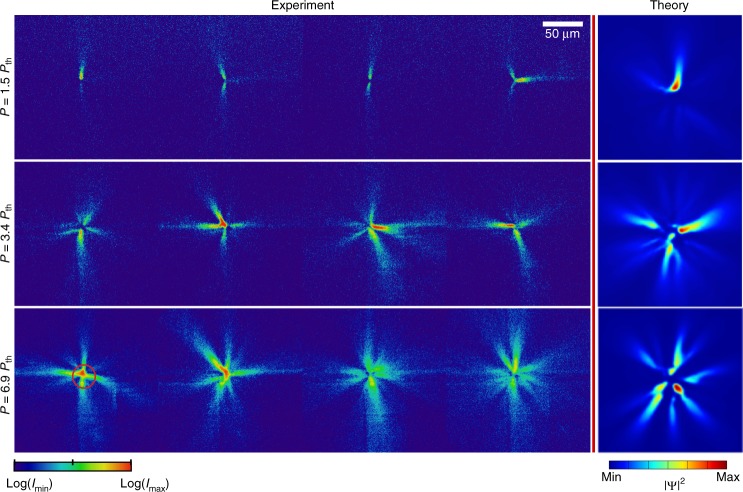
Single-shot condensation of photonic polaritons. The panels show single-shot real space images of the photoluminescence intensity (proportional to polariton density) above condensation threshold *P*_th_ ≈ 10 mW, in the far red-detuned (large negative detuning, Δ = −22 meV) regime for various pump powers. The circle indicates approximate location of the pump. Each panel represents a single realisation of a spontaneous condensation process. The intensity is plotted on a log scale for the experimental images to elucidate the details of regions with low density emission. The theory column shows polariton density obtained by numerical modelling

In our non-stationary condensation regime, the polaritons created by a short pulse just above the threshold power accumulate on top of the potential hill induced by the incoherent reservoir, which defines the offset (blue shift) of this state relative to *E*_min_(**k** = 0). The **k** ≠ 0, tails on the polariton dispersion arise due to ballistic expansion and flow of polaritons down the potential hill^48^, as shown schematically in Fig. 1(g). In the absence of appreciable phonon-assisted energy relaxation, this flow is non-dissipative and the initial blue shift is converted into the kinetic energy. Similar behaviour has been described in previous experiments in CW regime^32,40^. The latter experiments also demonstrated that, as the pump power grows, phonon-assisted relaxation into the ground state increases, leading to the transition to the energy and momentum ground state. Importantly, in the CW regime, the ground state condensate forms at the bottom of the potential hill formed by the pump-induced reservoir and is therefore spatially offset from the pump region^32^, as shown schematically in Fig. 1(h). The blue shift of this state from the minimum of the LP dispersion is purely due to polariton–polariton interaction, and is negligible for weakly interacting photon-like polaritons at large negative detunings. In striking contrast to these CW observations, our single-shot imaging of the condensate in real space shown in Fig. 2 and described below reveals that the condensate forms in the spatial region overlapping the long-lifetime reservoir. The absence of the reservoir-induced blue shift above condensation threshold for highly photonic polaritons, as shown in Fig. 1(d), is therefore puzzling and can be understood only by analysing the intricate details of the single-shot condensation dynamics, as discussed below.

### Single-shot condensation features

The single-shot real-space imaging reveals strong filamentation of the condensate at highly negative detunings (Δ <−14.5 meV) with filaments extending for macroscopic distances >100 μm, as seen in Fig. 2. The orientation of the filaments varies from shot-to-shot (see Supplementary Fig. 3 in Supplementary Note 2 for more detail), which rules out pinning of the condensate by a disorder potential in the microcavity^17^. Remarkably, filamentation of the condensate persists even when the ensemble-averaged dispersion shows clear spectral signatures of the bosonic condensation in a true ground state of the system (Fig. 1(d)), and the ensemble-averaged image of the spatial density distribution displays a smooth, nearly spatially homogeneous profile (Fig. 1(f), inset).

**Fig. 3:**
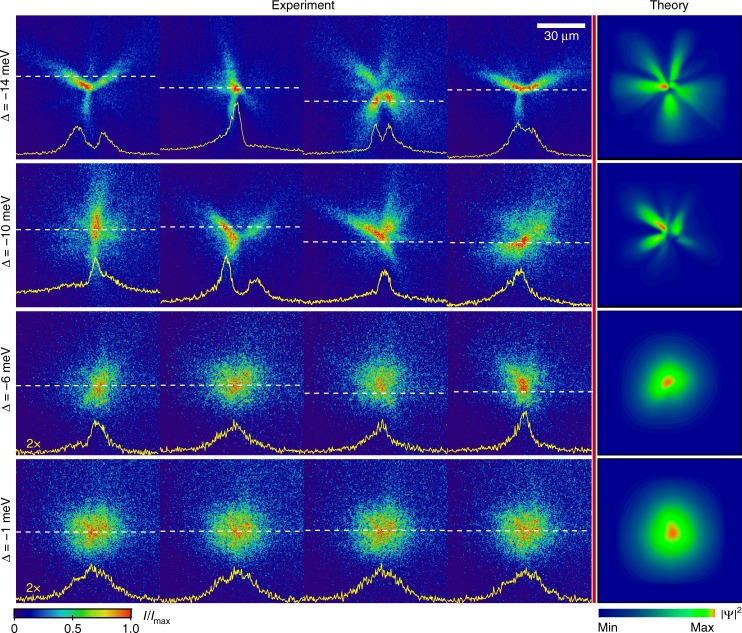
Single-shot condensation for various detunings. Single-shot real space images of the photoluminescence intensity (proportional to polariton density) for *P*/*P*_th_ ~ 6 and varying values of detuning Δ. Each panel represents a single realisation of spontaneous condensation and includes an image of the intensity profile at a cross-section marked by a dashed line. Intensity is plotted on linear scale. The theory column shows polariton density obtained by numerical modelling

**Fig. 4:**
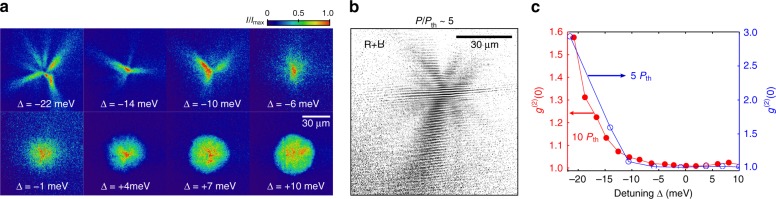
Spatial coherence measurements. **a** Representative single-shot real space images of the polariton density above the condensation threshold, *P*/*P*_th_ ~ 5, for a range of detuning values, Δ. **b** Ensemble-averaged interference of the condensate emission with its retroreflected image demonstrating the range of first-order spatial coherence, *g*^(1)^, extending across the entire condensate despite strong filamentation. The image is taken for Δ = −22 meV and *P*/*P*_th_ ~ 5. **c** Measurements of the second-order spatial density correlation function *g*^(2)^(0) for *P*/*P*_th_ ~ 5 and *P*/*P*_th_ ~ 10. Values of *g*^(2)^(0) > 2 indicate a non-Gaussian distribution of fluctuations

With increasing detuning, hence a larger excitonic fraction, we observe the transition to more smooth condensate profiles with reduced shot-to-shot density fluctuations. This transition is clearly seen in the real space images of the condensate presented in Figs. 3 and 4(a). Appreciable blue shift of the ground state due to polariton–polariton interactions is also seen for detunings Δ > −3 meV well above condensation threshold, *P*/*P*_th_ > 5 (see Supplementary Fig. 1 in Supplementary Note 1). In a more excitonic regime, the prevalence of the strong coupling in the pump region cannot be assured^49–51^ as the emission overlaps the cavity photon resonance at the early stages of the single-shot dynamics (see Supplementary Fig. 2 in Supplementary Note 1).

The measure of phase fluctuations in the system and therefore an indication of the long-range order (or absence thereof) is given by the first-order spatial correlation function *g*^(1)^, which can be deduced from the interference of the polariton emission in a single shot. Due to the highly inhomogeneous nature of spatial distribution, the measurement of *g*^(1)^(**r**, −**r**) in the retroreflected configuration relies on interfering two different filaments of the condensate that are shooting off in roughly opposite directions (details of this measurement are found in Supplementary Note 3). The typical interference pattern is shown in Fig. 4(b) for a highly photonic condensate with a high degree of filamentation. This measurement shows that the spatial coherence extends across the length of ~100 μm, which is comparable to the size of the whole condensate. Remarkably, this conclusion holds even for low pump powers above threshold, where only a few filaments are formed, and the condensate is highly spatially inhomogeneous. The long-range coherence maintained despite spatial fragmentation is reminiscent of the early condensation experiments affected by a disorder potential^3^.

To quantify the transition to a condensed state with reduced shot-to-shot density fluctuations, we calculate the zero-time-delay second-order density correlation function:

1 $$g^{(2)}\left( {\delta x,\delta y} \right) = \frac{{\left\langle {I\left( {x,y} \right)I\left( {x + \delta x,y + \delta y} \right)} \right\rangle }}{{\left\langle {I\left( {x,y} \right)} \right\rangle \left\langle {I\left( {x + \delta x,y + \delta y} \right)} \right\rangle }},$$

where *I*(*x*, *y*) is the camera counts at pixel position (*x*, *y*) of a single-shot image, and 〈〉 represents the ensemble average over the number of experimental realisations. This function is a measure of density fluctuations in the condensate. Since the single-shot images are time-integrated, the experimentally measured *g*^(2)^ function is a weighted average over the lifetime of the condensate. For second-order coherent light-matter waves *g*^(2)^(0, 0) ≡ *g*^(2)^(0) = 1, and for a condensate with strong density fluctuations *g*^(2)^(0) > 1^52^.

The measurement of *g*^(2)^(0) in our experiment is presented in Fig. 4(c), and demonstrates a clear transition from second-order incoherent polaritons for Δ <−5 meV to a second-order coherent polariton condensation regime. The *g*^(2)^(0) > 1 indicates that condensates of weakly interacting photon-like polaritons are characterised by large statistical fluctuations, which nevertheless coexist with macroscopic phase coherence as evidenced by the *g*^(1)^ (**r**, −**r**) measurement. Earlier experiments with short-lifetime polaritons support this conclusion^9^. Similar behaviour was recently observed for photon condensates strongly coupled to a hot reservoir, which acts both as a source of particles and a source of thermal fluctuations^53^ thus realising the grand-canonical statistical conditions. The apparent drop of *g*^(2)^(0) → 1 for condensates of more excitonic particles at larger detuning values then primarily indicates growth of the coherent condensate fraction in the system and depletion of the reservoir, as well as suppression of fluctuations due to increased interactions^54,55^.

### Theoretical modelling

To model the formation and decay of the condensate produced by a single laser pulse, we employ the driven-dissipative Gross-Pitaevskii model^34^ with a phenomenological energy relaxation responsible for the effective reduction of the chemical potential of the condensate and an additional stochastic term accounting for fluctuations ^48^:

2 $$i\hbar \frac{{\partial \psi \left( {\mathbf{r}} \right)}}{{\partial t}} = \left[ {\left( {i\beta - 1} \right)\frac{{\hbar ^2}}{{2m}}\nabla ^2 + g_{\mathrm{c}}\left| \psi \right|^2 + g_{\mathrm{R}}n_{\mathrm{R}} + i\frac{\hbar }{2}\left( {Rn_{\mathrm{R}} - \gamma _{\mathrm{c}}} \right)} \right]\psi \left( {\mathbf{r}} \right) + i\hbar\frac{{{\rm d}W}}{{{\rm d}t}},$$

3 $$\frac{{\partial n_{\mathrm{R}}\left( {\mathbf{r}} \right)}}{{\partial t}} = - \left( {\gamma _{\mathrm{R}} + R\left| {\psi \left( {\mathbf{r}} \right)} \right|^2} \right)n_{\mathrm{R}}\left( {\mathbf{r}} \right) + P\left( {\mathbf{r}} \right).$$

In Eq. (2), *R* defines the stimulated scattering rate, *γ*_c_ and *γ*_R_ are the decay rates of condensed polaritons and the excitonic reservoir, correspondingly. Constants *g*_c_ and *g*_R_ characterise the strengths of polariton–polariton and polariton–reservoir interactions, respectively. The rate of injection of the reservoir particles, *P*(**r**), in Eq. (3) is proportional to the pump power, and its spatial distribution is defined by the pump profile.

The model equations in this form can be consistently derived within the truncated Wigner approximation^55,56^. The term proportional to d*W*/d*t* introduces a stochastic noise in the form of a Gaussian random variable with the white noise correlations:

4 $$\left\langle {{\rm d}W_i^ \ast {\rm d}W_j} \right\rangle = \frac{{\gamma _{\mathrm{c}} + Rn_{\mathrm{R}}\left( {r_i} \right)}}{{2\left( {\delta x\delta y} \right)^2}}\delta _{i,j}{\rm d}t,\quad \quad \left\langle {{\rm d}W_i{\rm d}W_j} \right\rangle = 0,$$

where *i*, *j* are discretisation indices: **r**_*i*_ = (*δx*, *δy*)_*i*_. We note that both the loss and the gain, *γ*_*c*_ and *Rn*_R_, contribute to this term^48,55^. A single-shot realisation of the spontaneous condensation experiment thus corresponds to a single realisation of the stochastic process modelled by Eqs. (2, 3).

Importantly, the model parameters are varied consistently with the characteristic values for long-life polaritons at various values of the exciton–photon detuning. Specifically, we can estimate the values of the interaction coefficients *g*_c_ and *g*_R_ from the corresponding nonlinear part of the photon–exciton interaction Hamiltonian re-written in the basis of the lower and upper polariton states, $\hat \psi _{{\mathrm{LP}}} = C\hat \phi + X\hat \chi$ and $\hat \psi _{{\mathrm{UP}}} = X\hat \chi - C\hat \phi$, where *C* and *X* are the real-valued Hopfield coefficients^6^. When the cavity is excited by linearly polarised light (see, e.g., ref.^57^), *g*_c_ = *g*_ex_|*X*|^4^ and *g*_R_ = *g*_ex_|*X*|^2^. Here *g*_ex_ = (*α*_1_ + *α*_2_)/2 is exciton–exciton interaction strength, which is the sum of the triplet and singlet contributions (typically *α*_2_ ≪ *α*_1_), and we have neglected the saturation of the exciton interaction strength^6^. The absolute value of the triplet interaction coefficient is difficult to determine and is debated^31^. Here we assume the standard value $\alpha _1 = 6E_0a_{\mathrm{B}}^2$, where *E*_0_ is the binding energy of the Wannier–Mott exciton, and *a*_B_ is the exciton Bohr radius in the particular semiconductor^58,59^. For GaAs QW microcavities used in our experiments, *a*_B_ ≈ 7 nm and *E*_0_ ~ 10 meV. The Hopfield coefficient, which defines the value of the excitonic fraction, depends on the exciton–photon detuning as follows: $\left| X \right|^2 = \left( {1/2} \right)\left( {1 + {\mathrm{\Delta }}/\sqrt {4\hbar ^2{\mathrm{\Omega }}^2 + {\mathrm{\Delta }}^2} } \right)$, where 2ℏΩ is the Rabi splitting. Furthermore, the LP effective mass and decay rate for polaritons are also detuning-dependent via the Hopfield coefficients: 1/*m* = |*X*|^2^/*m*_ex_ + (1 − |*X*|^2^)/*m*_ph_, *γ*_c_ = |*X*|^2^*γ*_ex_ + (1 − |*X*|^2^)*γ*_ph_, which affects the respective parameters in the model equation. Finally, we assume that the stimulated scattering rate from the reservoir into the polariton states is more efficient for more excitonic polaritons: *R* = *R*_0_|*X*|^2^.

The phenomenological relaxation coefficient, *β*, defines the rate of the kinetic energy relaxation due to the non-radiative processes, such as polariton–phonon scattering, and is critical for modelling the highly non-equilibrium, non-stationary condensation dynamics presented here. This parameter is usually assumed to depend on the polariton^60^ or reservoir^26^ density; however, we find that the effect of the increasing detuning (from negative to positive) on growing efficiency of energy relaxation towards low-momenta states in our experiment is adequately described by increasing the value of the relaxation constant *β* ∝ |*X*|^2^.

The excellent agreement between the numerical simulations and experiment can be seen in real-space images shown in Figs. 2 and 3. Importantly, the filamentation effect observed in the experiments is reproduced in numerical simulations using the detuning-dependent parameters as described above. It is also critical to note that the initial condition for the simulations is the white noise *ψ*_0_, which essentially ensures that a non-stationary polariton mean-field inherits strong density and phase fluctuations^37^, because neither the reservoir nor the polariton density reach a steady state in our experiments.

**Fig. 5:**
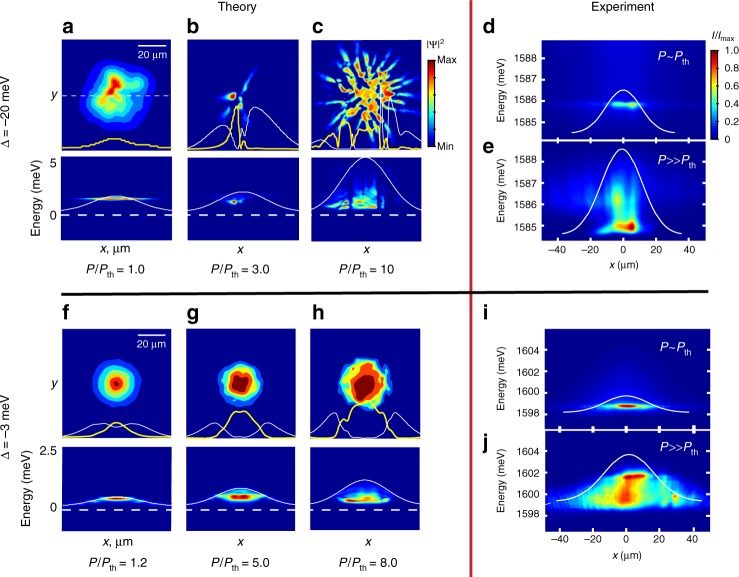
Theoretical results and spatially resolved energy measurements. **a**–**c**, **f**–**h** Numerically calculated single-shot real space density and corresponding lifetime-integrated real space spectra *E*(*x*) near and above the condensation threshold, for **a**–**c** highly photonic and **f**–**h** more excitonic polaritons. White and yellow curves in the single-shot density plots are the cross-sections of the reservoir (*n*_R_) and condensate (|*ψ*|^2^) densities, respectively. The |*ψ*|^2^ in **a** is scaled up by a factor of 30, *n*_R_ is off the scale and is not shown. White solid curves in *E*(*x*) plots are the initial blue shifts, *E*_R_, due to a non-depleted reservoir density *n*_R_. Dashed lines correspond to the minimum of the LP dispersion. **d**, **e** and **i**, **j** Experimentally measured, time-integrated and ensemble-averaged spectra *E*(*x*) for the (**d**, **e**) photonic (Δ = −20 meV) and **i**, **j** more excitonic (Δ = −3 meV) polaritons. Panels **d**, **i** and **e**, **j** correspond to the at and above threshold regimes, respectively, which are similar to the points **b** and **d** in Fig. 1(f). White solid curves show non-depleted reservoir density *n*_R_ deduced from numerical calculations

The model Eqs. (2 and 3) allow us to simulate the process of the condensate formation and dynamics after an initial density of reservoir particles is injected by the pump, and to reproduce the spatial and spectral signatures of the condensate in the different regimes shown in Figs. 2, 3. Importantly, using this model, we can investigate the features of the condensate formation near the threshold, where the densities are too low to be captured by the single-shot real-space imaging in our experiments. The simulations show that the condensate formation is seeded in one or several randomly located hot spots, which then locally deplete the reservoir at the spatially inhomogeneous rate proportional to the condensate density and the stimulated scattering rate *γ*_D_ = *R*|*ψ*|^2^. This depletion remains insignificant just below and at threshold, which leads to the bulk of polariton emission originating from the high-energy states on top of the reservoir-induced potential hill, which are blue-shifted from *E*_min_(**k** = 0) by the value of *E*_R_ = *g*_R_*n*_R_ (see Fig. 5(a)) and result in the emission shown in Fig. 5(d). Once the condensate forms at a particular hot spot, the *γ*_D_ at this location dramatically increases, and the condensate becomes trapped in local reservoir-induced potential minima, leading to spatial filamentation seen in the real space images Figs. 2 and 5(b, c). The location and size of the local trapping potentials is random at each realisation of the condensation process, which leads to large shot-to-shot density variations. The sharp filaments forming in this regime can be attributed to the effective self-focusing. Indeed, the reservoir depletion results in an effective saturable nonlinearity, which leads to attractive contribution to the repulsive mean-field interaction near condensation threshold^34,35,61^ (see Supplementary Note 4). This contribution dominates the mean-field interaction for highly photonic polaritons $\left( {\left| X \right|^2 \ll 1} \right)$, so that the effective attractive nonlinearity, characterised by *g*_eff_ ∝ *g*_c_(1−|*X*|^−2^), leads to very strong self-focusing of the filaments at the onset of condensation.

The spectral signatures of this regime shown in Figs. 1(e) and 5(b, c) confirm the scenario of the hole burning accompanied by energy relaxation (Fig. 1(h)), as seen in the experimental images Figs. 1(d), and 5(d, e). We note that the apparent narrowing of the energy trace with increasing pump power seen in Fig. 1(d) (as compared to Fig. 1(c)) is associated with the faster depletion rate *γ*_D_ due to the larger polariton density created by the stronger pump.

As the excitonic fraction in a polariton increases with growing detuning, phonon-assisted energy relaxation becomes more efficient and tends to suppress high-momentum excitations. This tendency is well captured by the phenomenological relaxation in our model, which provides damping of the (spatial) spectral components at the rate $\gamma _{{\mathrm{rel}}}\sim \hbar \beta \left| \bf{k} \right|^2/m$. The suppression of high-*k* fluctuations of density results in large area hot spots and a larger area of reservoir depletion right at the onset of condensation, Fig. 5(f). The spectral signatures of condensation in this regime, Fig. 5(g, h), qualitatively agree with the experimental images shown in Fig. 5(i, j). In addition, the self-focusing effect is much weaker due to reduced attractive correction to the effective nonlinearity (see Supplementary Note 4). This leads to the formation of condensates without dramatic filamentation and, at higher pump powers, complete phase separation between the condensate and the reservoir due to the depletion process, as shown in Fig. 5(g, h). We stress that such a dramatic hole burning effect due to irreversible reservoir depletion is not possible in a CW excitation experiment, where the reservoir is continuously replenished by a pump laser. The lack of spatial overlap between the condensate and the thermal reservoir leads to reduced statistical fluctuations^55^, as observed in Fig. 4(c). Since our measurement is integrated over the duration of the condensate life cycle, a value close to *g*^(2)^(0) = 1 indicates that the condensate quickly reaches a coherent stage and remains coherent as it decays. Due to the largely depleted reservoir, no revival of the thermal emission^39^ occurs as the condensate decays.

## Discussion

The remarkable agreement between our theory and single-shot experimental results unambiguously links transition to the spontaneous condensation in low energy and momenta states to the combination of two processes: energy relaxation, represented in our model by the rate *γ*_rel_ and local reservoir depletion characterised by *γ*_D_. As long as both of these rates are greater than the rate of the polariton decay *γ*_c_, the hole burning and efficient energy relaxation drive condensation to the ground state. Near threshold, the condensate growth rate $\gamma _{{\mathrm{cg}}}\sim Rn_0$, where *n*_0_ is the initial density of the reservoir injected by the excitation pulse, is also competing with the rate of ballistic expansion of polaritons due to the interaction with the reservoir. The latter is determined by the velocity of the polaritons acquired as the interaction energy with the reservoir *E*_R_ = *g*_R_*n*_R_ is converted to kinetic energy, and can be estimated as $\gamma _{{\mathrm{exp}}}\sim \left( {1/L} \right)\sqrt {2E_{\mathrm{R}}/m}$, where *L* is the spatial extent of the pump-reservoir region. Inefficient energy relaxation, and fast growth of the condensed fraction, *γ*_cg_ > *γ*_exp_, leads to large density fluctuations and condensation in several spatially separated filaments driven by the hole burning. The above scenario is realised in our experiment for large negative detuning, i.e., for largely photonic polaritons. In contrast, efficient energy relaxation and lower reservoir densities (pump powers) required for condensation lead to the rates of ballistic expansion being comparable to that of condensate growth, which results in more homogeneous condensate density. This scenario is realised for more excitonic polaritons at small negative and near-zero detunings.

Our results have several important implications for polariton physics. First, they offer a striking demonstration of the strong role of the reservoir depletion on the formation of a polariton condensate in the non-equilibrium, non-stationary regime. This demonstration is uniquely enabled by the single-shot nature of our experiment which ensures that, once depleted, the reservoir is never replenished: the reservoir which feeds the polariton condensate is created by a laser pulse and is depleted and/or decays before the next excitation pulse arrives. For polaritons with a high admixture of particle (exciton) component, i.e. in the regime when both reservoir depletion and phonon-assisted energy relaxation dominate the condensation dynamics, we are therefore able to create high-density condensates that are spatially separated from the reservoir, the latter acting as both a source of polaritons and a source of strong number (density) fluctuations. Such condensates, apart from demonstrating macroscopic phase coherence, exhibit second-order spatial coherence.

Secondly, our results indicate that spatial filamentation, similar to that attributed to dynamical instability of the polariton condensate in an organic microcavity^33^, is an inherent feature of the polariton condensation process, which is completely masked by any statistically averaging measurement and can only be uncovered in the single-shot regime. The filamentation arises due to the random spatial fluctuations inherited from the incoherent reservoir at the onset of the condensation. Efficient energy relaxation is critical for gradual suppression of these fluctuations with growing exciton–photon detuning, and subsequent formation of condensates with a reduced degree of filamentation.

The formation of filaments can be interpreted as self-focusing of the polaritons due to effectively attractive nonlinear interactions produced by the hole-burning effect at the early stages of the condensate formation. Although the hole burning has been implied in most conventional theoretical models of the polariton condensation under non-resonant optical excitation conditions^34^, here we present the direct observation of this effect and the associated self-focusing. However, we would like to point out that the self-focusing effect is not equivalent to modulational (dynamical) instability, the latter also present in other nonlinear matter-wave systems^62,63^. Indeed, as discussed in Supplementary Note 4, the hole burning and the associated effectively attractive nonlinearity is a necessary, but not sufficient, condition for instability of the steady-state polariton condensate in response to spatial density modulations predicted theoretically^34,35^ and observed experimentally^61^ in the CW regime. The transition to condensation at **k** = 0 observed in our experiments, even in the presence of filamentation, is also in contrast to the dynamical instability which is typically accompanied by spectral broadening^64^.

Last but not least, the single-shot imaging technique is a powerful tool for further studies of fundamental properties of the non-equilibrium condensation process, such as development of the macroscopic phase coherence in a polariton condensate strongly coupled to the reservoir. In particular, combination of the first-order correlation measurements and direct imaging of phase defects in the single-shot regime could assist in testing the Kibble–Zurek-type scaling laws in driven-dissipative quantum systems^37^.

## Methods

### Sample

The high Q-factor microcavity sample used in this work consists of twelve 7-nm GaAs QWs embedded in a 3*λ*/2 microcavity with distributed Bragg reflectors composed of 32 and 40 pairs of Al_0.2_Ga_0.8_As/AlAs *λ*/4 layers; similar to the one used in ref.^40^. The Rabi splitting is 2ℏΩ = 14.5 meV, and the exciton energy at normal incidence pumping is *E*_ex_(**k** = 0) = 1608.8 meV. The effective mass of the cavity photon is *m*_ph_ ≈ 3.9 × 10^−5^ *m*_e_, where *m*_e_ is a free electron mass.

### Experiment

We used a photoluminescence microscopy setup typical of the off-resonant excitation experiments with QW exciton polaritons in semiconductor microcavities. A single 50× objective (NA = 0.5) is used to focus the pump laser and collect the photoluminescence from the sample. The excitation energy is tuned far above (~100 meV) the polariton resonance to ensure spontaneous formation of the polariton condensate. The single-shot imaging is realised by employing a home-built high contrast ratio (~1:10,000) pulse picker that picks a single 140 fs pulse from a 80-MHz mode-locked Ti:Sapphire laser (Chameleon Ultra II). It is synced, using a delay generator SRS DG645, to an Electron-Multiplying CCD camera (Andor iXon Ultra 888) which is exposed for at least 10 μs before and after the pulse. The camera therefore records photoluminescence from the sample, which is integrated over the entire lifetime of the condensate and reservoir during a single realisation of the condensation experiment. Each single-shot real-space image is a time-integrated real-space distribution of polaritons in a single realisation of the condensation experiment. Experimental images taken without pulse picking result in ensemble-averaging over 10^6^ pulses. To reduce sample heating, the pulse train is chopped using an AOM at 10 kHz and 10% duty cycle.

The long lifetimes of polaritons allow for larger polariton densities to be accumulated above condensation threshold at a particular pump power compared to the short-lifetime samples, which leads to a brighter emission detectable in a single shot. A simple rate equation model based on the model Eqs. (2 and 3) allows us to construct a quantitative argument to support this statement. The reservoir density injected by the pump at condensation threshold can be estimated as $n_0\sim \gamma _{\mathrm{c}}/R$. The long lifetime therefore allows for smaller reservoir density to be injected by the pump, resulting in a lower threshold rate *P*_th_ *γ*_R_*n*_0_. At a given pump rate, *P* > *P*_th_, the polariton density can be estimated as $n_{\mathrm{c}}\sim \left( {\gamma _{\mathrm{R}}/R} \right)\left( {P/P_{{\mathrm{th}}} - 1} \right)$, and will be higher for longer-lifetime polaritons. This argument can explain the order of magnitude larger increase in the photoluminescence (PL) intensity above threshold for long-lifetime polaritons observed in our experiments as compared to the short-lifetime samples. Indeed, the early works on polariton condensation in microcavities with short lifetime polaritons, e.g. ref.^3,4^, reported the growth of the PL intensity above the threshold of 1–2 orders of magnitude. In contrast, in our sample (see Fig. 1(f) and ref.^31^), the growth of the PL intensity is at least 3 orders of magnitude.

### Data availability

The data that support the findings of this study are available from the corresponding author upon reasonable request.

## Electronic supplementary material

Supplementary Information

Description of Additional Supplementary Information

Supplementary Movie
