# Publisher Correction: Single-shot condensation of exciton polaritons and the hole burning effect

**DOI:** 10.1038/s41467-018-06064-w

**Published:** 2018-08-28

**Authors:** E. Estrecho, T. Gao, N. Bobrovska, M. D. Fraser, M. Steger, L. Pfeiffer, K. West, T. C. H. Liew, M. Matuszewski, D. W. Snoke, A. G. Truscott, E. A. Ostrovskaya

**Affiliations:** 1 ARC Centre of Excellence in Future Low-Energy Electronics Technologies and Nonlinear Physics Centre, Research School of Physics and Engineering, The Australian National University, Canberra, ACT 2601 Australia; 2 Institute of Molecular plus, Tianjin University, 300072 Tianjin, China; 3 Institute of Physics, Polish Academy of Sciences, A. Lotiników 32/46, 02-668 Warsaw, Poland; 4 JST, PRESTO, 4-1-8 Honcho, Kawaguchi, Saitama 332-0012 Japan; 5 Quantum Functional System Research Group, RIKEN Center for Emergent Matter Science, 2-1 Hirosawa, Wako-shi, Saitama 351-0198 Japan; 6 Department of Physics and Astronomy, University of Pittsburgh, Pittsburgh, PA 15260 USA; 7 Department of Electrical Engineering, Princeton University, Princeton, NJ 08544 USA; 8 Princeton Institute for the Science and Technology of Materials, Princeton University, Princeton, NJ 08544 USA; 9 Division of Physics and Applied Physics, School of Physical and Mathematical Sciences, College of Science, Nanyang Technological University, Singapore, 637371 Singapore; 10 Laser Physics Centre, Research School of Physics and Engineering, The Australian National University, Canberra, ACT 2601 Australia

Correction to: *Nature Communications* 10.1038/s41467-018-05349-4; published online: 9 August 2018

The original PDF version of this Article had an incorrect Published online date of 25 December 2018; it should have been 9 August 2018. This has been corrected in the PDF version of the Article. The HTML version was correct from the time of publication.

