# Supplementary material for: Single-shot condensation of exciton polaritons and the hole burning effect
Source: Nat Commun. 2018 Aug 9;9:2944. doi: 10.1038/s41467-018-05349-4 (PMC6085311; doi:10.1038/s41467-018-05349-4)
Supplement: Supplementary file 1 — Supplementary Information [file 41467_2018_5349_MOESM1_ESM.pdf]

Supplementary Information

Estrecho *et al.*, Single-shot condensation of exciton polaritons and the hole burning effect.

Supplementary Note 1. Dispersion measurements for various detunings.

The momentum- and energy-resolved photoluminescence (PL) as a function of pump power (similar to Figure 1(a-d) of the main text) for various values of the exciton-photon detuning Δ is shown in Supplementary Figure 1. These images are taken using a spatial filter (D~80 μm) in the real space image plane. The PL signal is time-integrated over the duration of a single-shot excitation and averaged over 10^6 realisations of the experiment.

In all cases shown ($\Delta < 0$ meV), we observe a blueshift of $\mathbf{k}\sim 0$ polaritons with the pump power below the condensation threshold P_{th} [1]. We define P_{th} as the pump power for which we observe both nonlinear growth of the PL intensity and abrupt narrowing of the linewidth. Above threshold, there is a drop in energy of $\mathbf{k}\sim 0$ polaritons towards the bottom of the lower polariton dispersion and further overall blue shift with increasing pump power. The resulting effect is smearing out of the time-integrated image along the energy axis. At pump powers $P/P_{\text{th}} < 10$ and $\Delta < 0$ meV, the system is in the strong coupling regime because it does not follow the cavity photon dispersion, and therefore exciton-polariton condensation is observed.

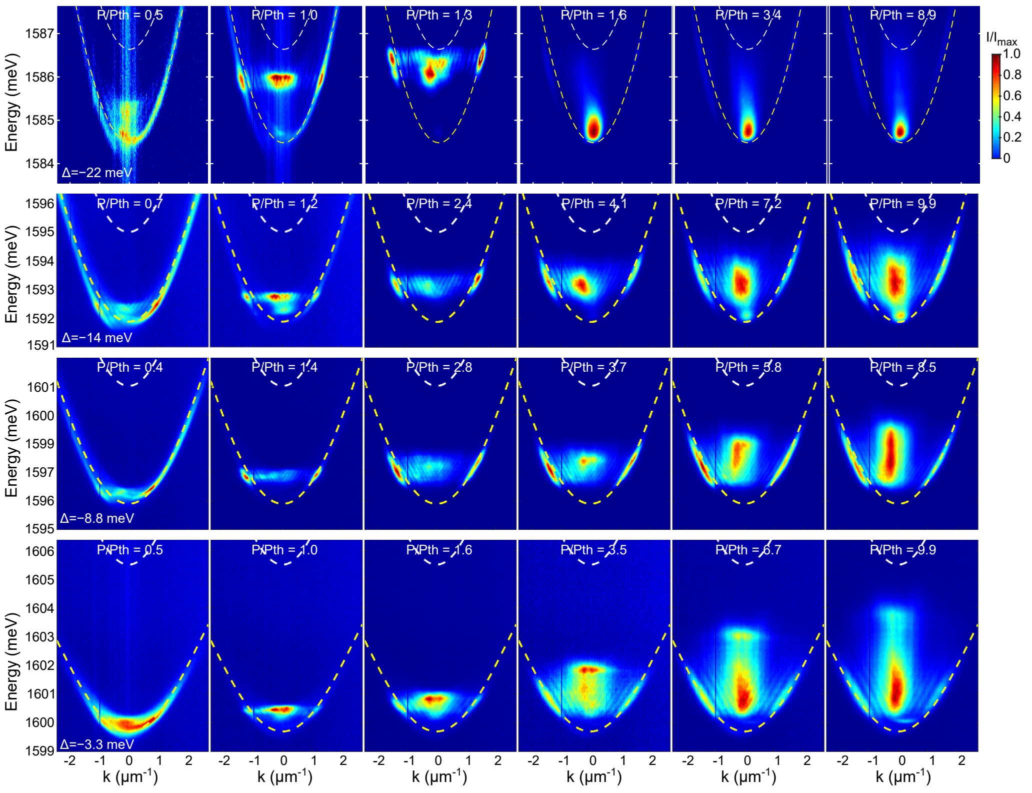

Supplementary Figure 1. Dispersion measurements for photonic polaritons. Time- and ensemble-averaged images for various detunings and pump powers are taken without pulse picking. The dashed white (yellow) curves correspond to the cavity photon (lower polariton) dispersion.

At near-zero and positive detunings, the blue shift can be large even at moderate pump power and can become comparable to the cavity photon energy. The overlap with the cavity photon

resonance may indicate loss of the strong coupling in the microcavity at these pump powers and detunings. A typical dispersion demonstrating this behavior is shown in Supplementary Figure 2 for $\Delta \approx 5$ meV. In this regime, during the initial stages of the single-shot dynamics, we might be observing a combination of photon lasing and polariton condensation.

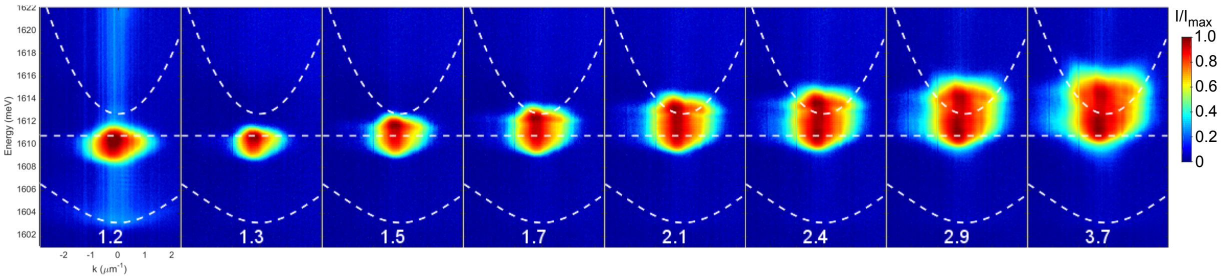

Supplementary Figure 2. Dispersion measurements for more excitonic polaritons. The images are taken without pulse picking at $\Delta \approx 5$ meV. The dashed curves at high and low powers correspond to the cavity photon and lower polariton dispersions, respectively. The dashed line corresponds to the exciton dispersion. The numbers in each panel indicate the pump power relative to the condensation threshold, $P/P_{\rm th}$.

Supplementary Note 2. Single-shot images for various pump powers and detunings.

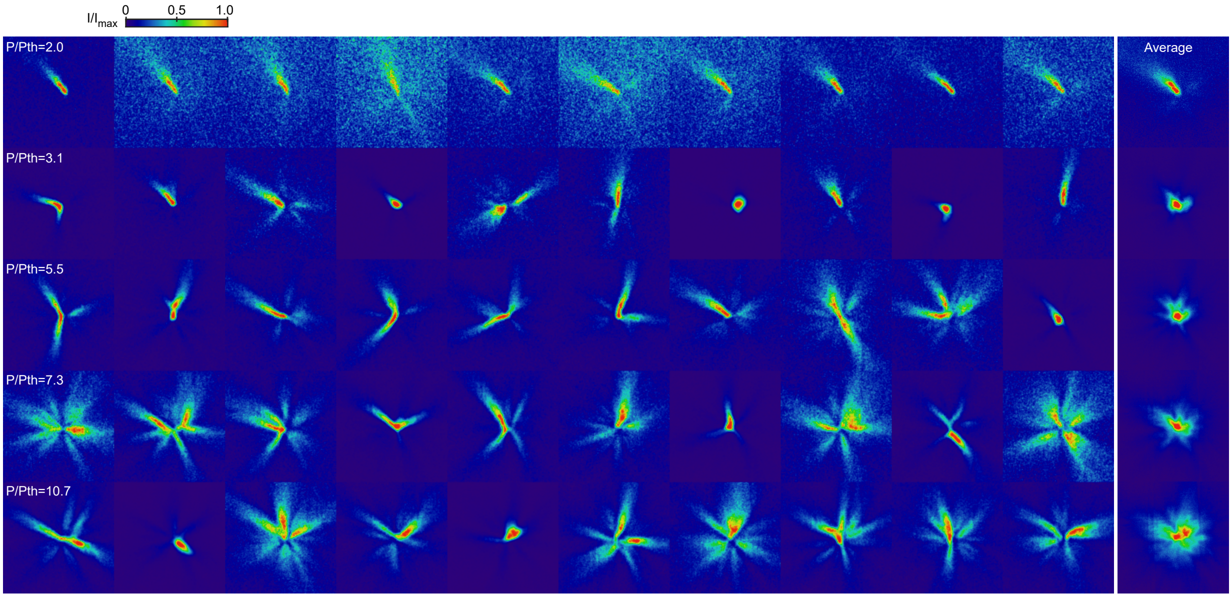

Supplementary Figure 3. Single-shot condensation of photonic polaritons. Real space single-shot images (90×90 μm^2) of the PL intensity, I, (proportional to the polariton density) taken at the detuning value $\Delta = -22$ meV. Each row represents different condensate relisations for the same pump power, and the average image represents ensemble averaging over $n = 25$ shots. Color scale is linear and each image is normalized.

Here, we describe the spatial properties of the single-shot images for different detuning and pump power. For the highly fragmented condensate ($\Delta = -22$ meV), we observe a single

filament just above threshold ($P/P_{\text{th}} = 2.0$) that is oriented in one direction (see Supplementary Figure 3). As we increase the pump power, we see the emergence of more filaments with shot-to-shot variation, as discussed in the main text.

It is important to note that the image averaged over 25 realisations of the experiment is not completely smooth, i.e. a dominant orientation of the filaments can be seen in Supplementary Figure 3, especially at lower pump power. To some extent, this is true even for the images averaged over 10^6 shots. We attribute this preference in direction to the wedge (change of the length) in the cavity, which creates a steep potential slope [1] for the polaritons, and to spatial inhomogeneity of the reservoir due to intensity variation across the pump profile. The latter effect dominates when the pump power increases as can be seen in Supplementary Figure 3. These effects create conditions that favor certain filament orientation, thus creating the dominant filaments on an averaged image.

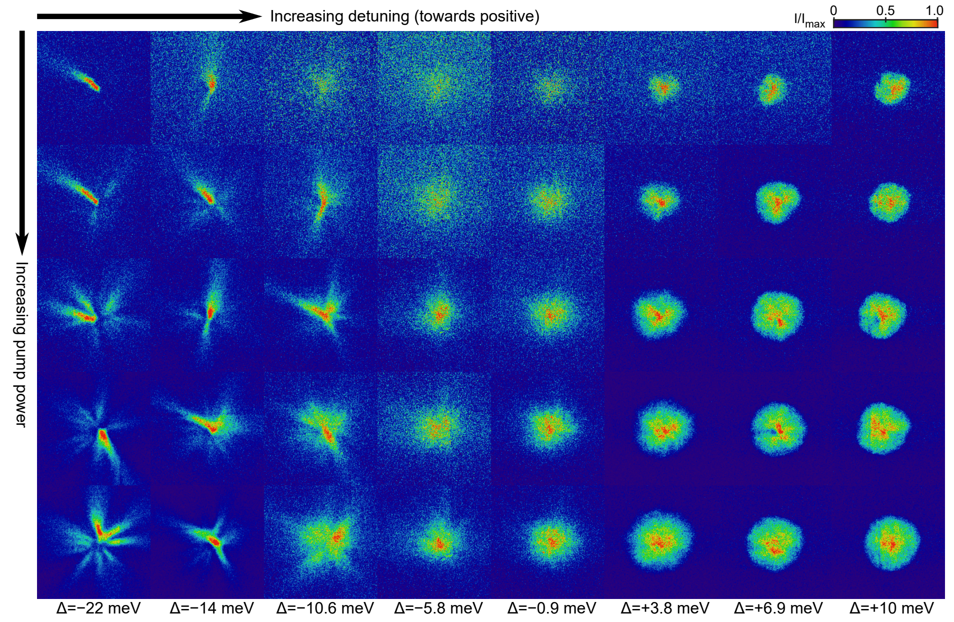

Supplementary Figure 4. **Single-shot condensation for various detunings.** Representative single-shot real-space images (90×90 μm^2) of the PL intensity shown as a function of pump power above threshold and exciton-photon detuning. Each image is normalised. The corresponding **Supplementary Video** shows random shot-to-shot variations for each of the images in this figure.

The overall trend of the single-shot images with varying detuning and pump power is shown in Supplementary Figure 4 and the corresponding Supplementary Video. In general, as the pump power is increased, the overall size of the condensate grows. There are two distinct regimes: (1) highly fragmented condensates forming thin filaments extending over large distances at highly negative detuning, and (2) spatially smooth, compact condensates forming in the pump region at small negative and positive detuning. Density fluctuations in the latter regime are suppressed, which results in perfectly smooth real-space images when averaged over 10^6 realisations of the single-shot experiment.

Supplementary Note 3. $g^{(1)}$ measurement for highly fragmented condensates

Long-range coherence. To measure the first order spatial correlation function $g^{(1)}$of highly spatially fragmented condensates, we employ a Michelson interferometer, which interferes a retroreflected real-space image with itself. The goal is to measure the spatial coherence between different filaments of the same shot. Due the shot-to-shot variation of the filament orientation, fringes appear in isolated regions in space, where the filaments of the two images interfere. These regions vary from shot-to-shot as well, since the filament orientation is random. When many single-shot interference images are averaged, the visibility of the fringes is dramatically reduced, which results in underestimation of the value of $g^{(1)}$. This is in addition to the effect of time-integration of the whole condensate dynamics over the single-shot experiment duration, which can wash out the fringes. In this case, a nonvanishing visibility on an averaged image implies a high degree of coherence.

Supplementary Figure 5 shows a time-integrated, ensemble-averaged interferogram of the condensate at a highly negative detuning. Here, we are able to interfere dominant filaments (aligned along the white arrows) of the condensate. These filaments have higher chance of appearing in single-shot images and therefore the fringe visibility survives when averaged. To normalise the interferogram and remove the spatial density variation, we used the formula given in the inset of Supplementary Figure 5(c) [2]. Here, taking a line profile along the filament direction shown in cyan arrow of Supplementary Figure 5(b), we obtain a clear evidence of the long-range spatial coherence extending to $\Delta y = 100$ μm (Supplementary Figure 5(c)), which is limited only by the signal-to-noise ratio of this particular measurement.

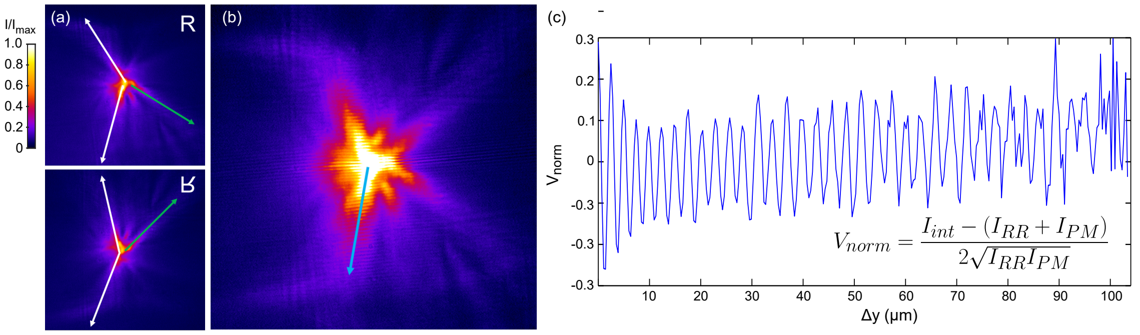

Supplementary Figure 5. **Measurement of the degree of spatial coherence.** (a) Interferogram used to measure the degree of spatial coherence for different filaments in a highly negative detuning regime. Shown are the plane mirror reflected image I_{PM} (marked with R) and the corresponding retroreflected image I_{RR} showing the three prominent filaments (white and green arrows). (b) Interference image I_{int} of the images in (a) where there is good overlap between the prominent filaments (white arrows in (a)). (c) Normalised interferogram line profile along the cyan arrow in (b) showing the appreciable visibility at large separation distance. Inset: the formula used to normalise the interferogram. The images in (a) and (b) have the field size of 135×135 μm^2.

Phase distribution. The $g^{(1)}$ measurements presented above confirm the existence of the long-range coherence in the highly spatially fragmented condensate, i.e. the relative coherence between separate, spatially extended filaments. For the future experiments on spontaneous condensate formation using the single-shot technique, it would be important to also extract the spatial distribution of the phase. To this end, a Mach Zehnder interferometer with a spatial filter and magnification optics to use a single filament phase as a reference for the whole image (see, e.g., [3]). While such an experiment is beyond the scope of our present study, it will be an important step in detecting and tracing the phase domains and singularities arising in the process of the condensate formation. Our numerical simulations (see Supplementary Figure 6) suggest that the spatial phase distribution is indeed very complex, and that formation of spatial filaments is associated with a multitude of phase defects and vortices.

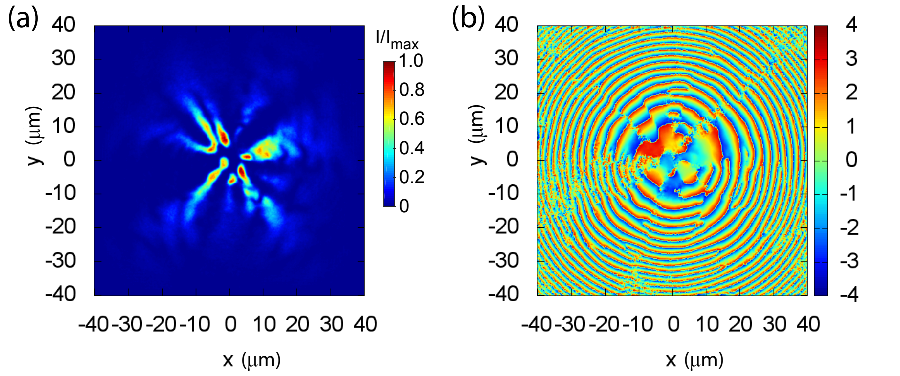

Supplementary Figure 6. **Typical instantaneous spatial distribution of the condensate.** (a) Polariton density and (b) phase, φ, in a spatially fragmented regime, as deduced from numerical simulations of the model Eqs. (2,3) of the main text. The field size in (a,b) is 80×80 μm^2, and the phase in (b) is wrapped. The complicated phase distribution in (b) is superimposed onto a radial phase gradient associated with the radial component of the condensate flow velocity $v_r \propto d\varphi/dr$.

Supplementary Note 4. Hole burning, effective interactions, and dynamical instability.

CW regime. Here we re-iterate the relationship between the hole burning (reservoir depletion) effect, the effective polariton-polariton interaction, and dynamical instability. For simplicity, we begin with the more conventional CW regime. In this regime, neglecting the energy relaxation and fluctuations in the model Eqs. (2,3) in the main text of the paper, one can obtain the stationary spatial density distribution of the reservoir particles:

$$n_{\mathrm{R}} = \frac{P}{\gamma_{\mathrm{R}} + R|\psi|^2}.$$

Clearly, larger condensate fraction leads to partial depletion of the reservoir density. In the limit of small condensate density (e.g., near condensation threshold), one can expand the above expression as follows:

$$n_{\mathrm{R}} \approx n_0 - n_0 \frac{R}{\gamma_{\mathrm{R}}} n_c + \cdots = n_0 - \frac{P}{P_0} \frac{\gamma_{\mathrm{c}}}{\gamma_{\mathrm{R}}} n_{\mathrm{c}} + \cdots$$

where we have introduced the notation: $n_c = |\psi|^2$, $n_0 = P/\gamma_R$, and $P_0 = \gamma_c\gamma_R/R$. Therefore, the presence of the reservoir introduces both a repulsive potential barrier proportional to n_0, and a correction to the polariton-polariton interaction energy, so that the effective polariton-polariton interaction constant in Eq. (2) of the main text can be re-written as follows:

$$g_{\text{eff}} = g_c\left(1 - \frac{g_R}{g_c}\frac{P}{P_0}\frac{\gamma_c}{\gamma_R}\right),$$

and can become negative. The latter means that the effective polariton-polariton interaction is attractive rather than repulsive if:

$$\frac{P}{P_0} > \frac{g_c}{g_R}\frac{\gamma_R}{\gamma_c}.$$

Under the additional assumption that the CW pump is spatially homogeneous, it is possible to derive an analytical condition for the onset of modulational (dynamical) instability [4]:

$$\frac{P}{P_0} < \frac{g_R}{g_c}\frac{\gamma_c}{\gamma_R},$$

where P_0 now has the physical meaning of the reservoir injection rate at condensation threshold P_{th}. Note that the two inequalities are different in general, but coincide exactly at threshold, where $P/P_0 = 1$. Given the dependence of the system's parameter on the excitonic Hopfield coefficient discussed in the main text, we can deduce that:

$$\frac{g_R}{g_c}\frac{\gamma_c}{\gamma_R} \approx \frac{1-|X|^2}{|X|^2}\frac{\gamma_{\text{ph}}}{\gamma_{\text{ex}}}$$

Assuming that the cavity photon lifetime in the high-Q microcavity is only an order of magnitude shorter than that of the exciton $\gamma_{\text{ph}}/\gamma_{\text{ex}} \sim 10$, we can obtain the diagram of effective interaction and instability domains shown in Supplementary Figure 7.

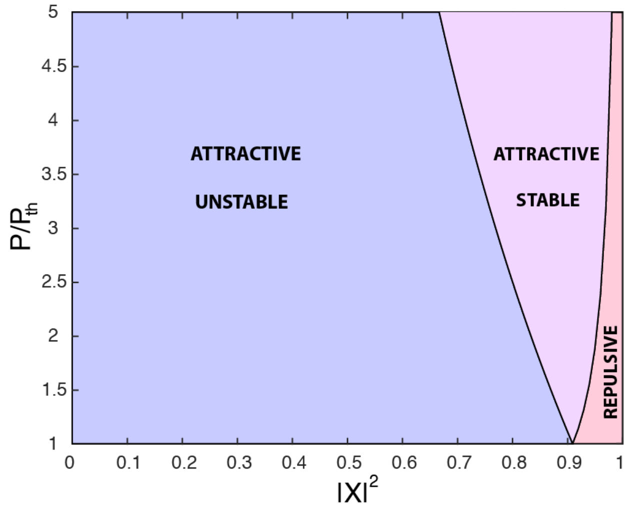

Supplementary Figure 7: **Domains of dynamical instability in CW regime.** Shown are stability/instability domains and the corresponding regimes of effectively attractive/repulsive interactions for a homogeneously pumped condensate.

Note that for largely excitonic polaritons there exists a region where, despite the influence of the reservoir, the effective nonlinearity is repulsive. This region disappears for the exciton polaritons in cavities with a lower Q-factor, and the instability boundary moves towards larger values of $|X|^2$.

Single-shot regime. The above analysis, while giving a general idea of the relationship between the different effects under consideration, is not applicable for the case of pulsed, single shot condensation regime. In this regime, once the initial density of the reservoir, n_0, is injected into the system at $t = 0$, the stationary density is never reached. The relationship between the condensate and reservoir densities can be derived from Eqs. (2,3) by neglecting the relaxation and fluctuations, and setting $P = 0$ for $t > 0$. If the rate of reservoir depletion is much grater than the rate of reservoir decay, then:

$$n_{\mathrm{c}}(t) \cong \frac{\gamma_{\mathrm{c}}}{R}\ln\left(1 - \frac{n_{\mathrm{D}}}{n_0}\right) + n_{\mathrm{D}},$$

where $n_{\mathrm{D}}(t) = n_0 - n_{\mathrm{R}}(t)$. Assuming, once again, that the condensate density is small and therefore the reservoir is weakly depleted, we obtain:

$$n_{\mathrm{R}}(t) \approx n_0 - \frac{n_0 R}{n_0 R - \gamma_{\mathrm{c}}} n_{\mathrm{c}}(t) + \cdots = n_0 - \frac{P}{P - P_0} n_{\mathrm{c}}(t) + \cdots,$$

where $P_0 \equiv P(t = 0) = n_0 \gamma_{\mathrm{R}}$. Under these assumptions, the effective interaction coefficient is time-independent and takes the form:

$$g_{\mathrm{eff}} = g_{\mathrm{c}}\left(1 - \frac{g_{\mathrm{R}}}{g_{\mathrm{c}}}\frac{P}{P - P_0}\right).$$

Given that $g_{\mathrm{R}}/g_{\mathrm{c}} = 1/|X|^2$, it follows that the effective interaction is always attractive ($g_{\mathrm{eff}} < 0$). For a spatially broad pump near the homogeneous threshold ($P_{\mathrm{th}} \to P_0$) the attractive interaction and associated self-focusing is very strong, as observed in [5]. For a more tightly focused pump ($P_{\mathrm{th}} \gg P_0$) the effective interaction constant is large and attractive for highly photonic polaritons, which results in strong self-focusing into sharp filaments as observed in our experiments. Conversely, the effective interaction constant tends to zero for highly excitonic polaritons, which results in a weaker self-focusing effect and spatially smooth condensates (see main text).

Supplementary References

[1] Nelsen, B., Liu, G., Steger, M., Snoke, D. W., Balili, R., West, K. & Pfeiffer, L. Dissipationless Flow and Sharp Threshold of a Polariton Condensate with Long Lifetime, Phys. Rev. X **3**, 041015 (2013).

[2] Bass, A., Lagoudakis, K. G., Richard, M., Andre, R., Dang, L. S. & B. Deveaud-Pledran, Synchronized and Desynchronized Phases of Exciton-Polariton Condensates in the Presence of Disorder, Phys. Rev. Lett. **100**, 170401 (2008).

[3] Manni, F., Lagoudakis, K. G., Liew, T. C. H., André, R. & Deveaud-Plédran, B. Spontaneous Pattern Formation in a Polariton Condensate, Phys. Rev. Lett. **107**, 106401 (2011)

[4] Smirnov, L. A., Smirnova, D. A., Ostrovskaya, E. A. & Kivshar, Y. S. Dynamics and stability of dark solitons in exciton-polariton condensates, Physical Review B **89**, 235310 (2014).

[5] Bobrovska, N., Matuszewski, M., Daskalakis, K. S., Maier, S. A. & Kéna-Cohen, S. Dynamical instability of a non-equilibrium exciton-polariton condensate, ACS Photonics, **5** 111 (2018).
