# Supplementary material for: Single-shot condensation of exciton polaritons and the hole burning effect
Source: Nat Commun. 2018 Aug 9;9:2944. doi: 10.1038/s41467-018-05349-4 (PMC6085311; doi:10.1038/s41467-018-05349-4)
Supplement: Supplementary file 2 — Description of Additional Supplementary Information [file 41467_2018_5349_MOESM2_ESM.pdf]

**Description of Additional Supplementary Files**

File Name: Supplementary Movie 1
Description: Supplementary Movie shows random shot-to-shot variations of polariton density in real space for each of the images in Supplementary Figure 4.
